# Attitudes among the general Austrian population towards neonatal euthanasia: a survey

**DOI:** 10.1186/1472-6939-15-74

**Published:** 2014-10-07

**Authors:** Lena Goldnagl, Wolfgang Freidl, Willibald J Stronegger

**Affiliations:** Institute of Social Medicine and Epidemiology, Medical University of Graz, Universitaetsstrasse 6, 8010 Graz, Austria

**Keywords:** Neonatal euthanasia, Attitude, Public opinion, Austria, End-of-life care, Groningen protocol

## Abstract

**Background:**

The Groningen Protocol aims at providing guidance in end-of-life decision-making for severely impaired newborns. Since its publication in 2005 many bioethicists and health care professionals have written articles in response. However, only very little is known about the opinion among the general population on this subject. The aim of this study was to present the general attitude towards neonatal euthanasia (NE) among the Austrian population and the factors associated with the respondents’ opinion.

**Methods:**

A cross-sectional study was conducted among the general Austrian population. Computer-assisted telephone interviews were performed with 1,000 interviewees aged 16 years and older. Binary logistic regression was performed in order to determine factors that are independently associated with the respondents’ opinion about neonatal euthanasia.

**Results:**

While 63.6% of the participants rejected the idea of neonatal euthanasia for severely impaired newborns, 36.4% opted either in favor or were undecided. Regression analysis has shown the respondents’ educational level (p = 0.005) and experience in the care of terminally ill persons (p = 0.001) to be factors that are positively associated with the rejection of neonatal euthanasia, whereas a higher age was associated with a lower degree of rejection (p = 0.021).

**Conclusions:**

We found that the majority of the Austrian population rejects the idea of neonatal euthanasia for severely impaired newborns. However, given the increasing levels of rejection of NE among the younger generations and among people with a higher educational level, it cannot be precluded that the rejection rate might in future increase even further, rather than decrease.

## Background

Voluntary active euthanasia (VAE) is a highly discussed topic throughout Europe. In the Netherlands VAE was legalized in 2002 for competent adults and minors from the age of 12 upwards [1]. This legislation, however, requires specific conditions to be fulfilled before a patient’s life can be ended: the request for euthanasia must be voluntary and carefully considered, the suffering must be unbearable, there must not be any other reasonable alternatives, an independent physician must have been consulted, and the request must be properly reported. In the case of minors, parental consent is additionally required. Only when all the above conditions are met are physicians exempted from criminal liability. Since the law makes no mention of newborns, neonatal euthanasia (NE) is still illegal. Nevertheless NE has been known to take place in the Netherlands [2–4].

After many years of open discussion, Verhagen and Sauer published the Groningen Protocol in 2005. It was developed at the University Medical Center of Groningen based on legal precedents and explicitly supported NE [5]. One of the main goals of the Groningen Protocol was to enable a more transparent end-of-life decision-making for newborns and to provide guidance on how to properly report these decisions. Neonates, for whom this protocol was intended, can be categorized into three groups. Group one consists of infants without any chance of survival despite receiving optimal medical treatment. This group comprises infants with a severe underlying disease, such as lung or kidney hypoplasia. The second group includes newborns who can only survive with intensive treatment and who will die shortly after the withdrawal of intensive care; for example infants with severe brain abnormalities or extensive organ damage caused by extreme hypoxemia. Lastly, there is the third and most controversial group, consisting of neonates who might survive in the long run but whose suffering is considered to be unbearable and impossible to alleviate. A highly typical example is a child with the most serious form of spina bifida [5, 6].

Furthermore, the Groningen Protocol lists several conditions that have to be fulfilled before a physician may attempt to end a newborn life. First of all, the doctor must be absolutely certain about both the diagnosis and prognosis for the newborn, and unbearable suffering must be present. Second, due to the lack of any decisional capacity, neonates are incapable of giving their consent. Therefore, the informed consent of both parents is required. Another requirement is that the diagnosis, prognosis, and unbearable suffering must be confirmed by at least one independent physician. Lastly, the procedure must be performed according to the accepted medical standard [5, 7]. Cases of NE are reviewed and where the tight guidelines are met, prosecutors will not bring a charge against the physician who carried out NE.

Since the publication of the Groningen Protocol, many bioethicists and health care professionals involved in the treatment of severely ill newborns have written in response and questioned its justification [8–14]. Supporters of NE argue that there are neonates whose suffering cannot be relieved, even when withdrawing the life-sustaining treatment, and for whom there is no hope of improvement. Their central argument is based on the judgment of the neonate’s quality of life, arguing that in such cases death would be more humane than a continued life. According to this reasoning, life-ending measures can be acceptable in such cases of unbearable suffering, if conducted under very strict conditions [5].

In Austria, active euthanasia is illegal for anyone, including newborn children. In recent years, we find a recurring public debate supporting either the liberalization of euthanasia for adults or the protection of the legal *status quo*. During the Nazi period, involuntary euthanasia programs were installed in Austria, directed at both mentally and physically disabled adults but also children [15]. Due to the historical burden, active euthanasia for neonates is a delicate subject in Austria that is neither discussed in public nor by the scientific medical community. Studies investigating the attitude toward NE among health professionals or lay people in Austria are lacking. Admittedly, only a small number of investigations on this topic can be found in international scientific literature. The EURONIC project is an important study conducted among the staff of neonatal intensive care units in several European countries. It presents the opinions of neonatologists and nurses on the diverse legal regulations and their self-reported practices for end-of-life decision-making in 10 different European countries [2, 16]. A French group investigating the attitude towards end-of-life decision making for newborns addressed which method of ending a newborn life is more acceptable among the French [17, 18]. They came to the conclusion that euthanasia was less accepted than withdrawing or withholding care. They suggested that acceptability was a function of the circumstances.

Very little is known about the general public attitude towards NE; only the two studies presented by Teisseyre et al. [17, 18] addressed the public, however, they were not based on representative samples. Therefore, the aim of this paper was to present the prevailing attitude towards NE as well as the factors associated with the respondents’ opinion among a representative sample of the Austrian population.

## Methods

### Study design, participants and data collection

The cross-sectional survey about attitudes toward euthanasia was conducted among inhabitants of Austria aged 16 years and older in December 2009. Participants were contacted via computer-assisted telephone interviews (CATI), a telephone surveying technique in which the interviewer is guided by a script provided through a software application. Telephone numbers were sampled from the current electronic telephone directory of Austria using the random-last-digits procedure (RLD). This allowed the inclusion of private and secret telephone numbers as well as of mobile phone numbers that are not listed in publicly available telephone directories. A randomized selection and screening procedure based on age, sex, and educational level was used to select interviewees from within contacted households. In order to complete a representative sample of 1,000 interviews 2,413 persons had to be contacted (response rate = 41.4%). This survey was conducted by the *Institute of Empirical Social Research* (IFES, Vienna) on behalf of the *Institute of Social Medicine and Epidemiology (Graz)*. To ensure representativeness of the final sample, IFES constructed a weighting variable based on representative values of the basic socio-demographic characteristics of the Austria population. Persons unable to communicate in German were excluded before starting the interview.

After calling a selected person, verbal informed consent was obtained from all individuals that were able and willing to participate in the study, otherwise calls were discontinued by the interviewer. All information that was entered into the survey was anonymous. Identification based on the provided data was impossible at any time. The Ethics Committee of the Medical University of Graz waived the necessity for ethical approval.

### Variables

*Age* was categorized into 6 different groups: 16–24, 25–34, 35–44, 45–59, 60–74, and 75 years and older. *Educational level* was divided into the following categories: ‘compulsory school’ (9 yrs of education), ‘apprenticeship/vocational school’ (10 to 12 yrs), ‘high school diploma’ (12 to 13 yrs) and ‘university diploma’ (15 yrs or more). Moreover, the interview included questions about the respondents’ *socio-cultural ideology* (‘conservative’ , ‘liberal’) and *political orientation* (‘left-wing’ , ‘center’ and ‘right-wing’). Additionally, interviewees were asked whether they had any *experience in the care of severely ill* (‘yes’ , ‘no’) or any *end-of-life care experience* (‘yes’ , ‘no’) and how they would self-rate their health (‘very good’ , ‘good’ , ‘moderate’ , ‘poor’ , ‘very poor’). Data on *marital status* as well as the *number of persons* and the *number of children* in the household were also collected.

The problem formulation specifically addressed the highly controversial group of infants included in the Groningen Protocol (with response categories ‘in favor’, ’against’, ‘undecided’ and ‘don’t know’). The question about the attitude towards NE was preceded by items concerning attitudes toward euthanasia for terminally ill adults. The wording of the NE item was:

“And now for another medical situation that refers to the beginning rather than to the end of life: *A new-born child is diagnosed with a serious illness or severe disability, leading to a life expectancy of only a few years in poor quality of life. In this case, are you personally in favor or against the administration of a lethal drug injection at child birth to spare the infant further suffering?*”

Answer categories were ‘approve’ , ‘disapprove’ , ‘undecided’ and ‘don’t know’. The categories ‘undecided’ and ‘don’t know’ were both interpreted as ‘depending on the circumstances’ and were therefore allocated to ‘approve’ in order to dichotomize the answer categories. To evaluate the attitude towards NE, we classified the answer categories into either ‘approve’ or ‘disapprove’, with disapprovers being the actual target group of our analysis. A similar approach was taken by Moulton et al. [19] and in a previous analysis performed with data of this survey [20].

### Data analysis

Univariate analyses were performed by cross-tabulating attitudes by determinants. Associations were tested using Chi^2^-tests for independence. Stepwise binary logistic regression was performed in order to determine factors independently associated with the respondents’ opinion on NE for severely impaired newborns. All analyses were adjusted for sex and age. Variables with p > 0.1 were excluded by backward procedure. We used a threshold value of 0.1 as exclusion criteria, which resulted in maintaining near-significant variables showing p-values between 0.05 and 0.1 in the model. Statistical analysis was carried out using IBM® SPSS Statistics 19.0 software for MS Windows® and statistical significance was defined as p ≤ 0.05.

## Results

### Univariate correlates of attitudes

The final sample of 1,000 persons (aged 16 to 90 years, mean age 46.3 years) comprised 473 men (47.3%) and 527 women (52.7%). 63.6% of all interviewees rejected NE while the other 36.4% (‘approvers’ by definition) included persons who opted in favor, were undecided, or didn’t answer the item (Table 1). No significant difference was found between men and women regarding their rejection rates.

**Table 1:** **Univariate analyses – attitudes toward neonatal euthanasia in per cent, by socio-demographic characteristics**

	Cases	Rejection	Approval	Chi ^2^-test
	N	%	%	P-value
**Total sample:**	1000	**63.6**	**36.4**	-
**Sex:**				
Male	473	62.8	37.2	0.630
Female	527	64.3	35.7	
**Age group:**				
16-25 yrs.	124	**76.0**	**24.0**	**>0.001**
25-34 yrs.	170	**70.6**	**29.4**	
35-44 yrs.	189	**70.4**	**29.6**	
45-59 yrs.	244	**57.4**	**42.6**	
60-74 yrs.	216	**58.8**	**41.2**	
75 years or older	57	**38.6**	**61.4**	
**Level of education:**				
Compulsory school	159	**48.4**	**51.6**	**>0.001**
Apprentice/vocational degree	543	**61.6**	**38.4**	
High school diploma	163	**72.4**	**27.6**	
University	127	**80.3**	**19.7**	
**Income:**				
1.quintile	208	66.8	33.2	0.690
2.quintile	185	64.3	35.7	
3.quintile	193	63.7	36.3	
4.quintile	203	59.8	40.2	
5.quintile	184	63.8	36.2	
**Socio-cultural ideology:**				
Conservative	314	63.1	36.9	0.653
Liberal	600	64.6	35.4	
**Political orientation:**				
Left-wing	222	**67.7**	**32.3**	**0.032**
Centre	521	**66.0**	**34.0**	
Right-wing	144	**55.2**	**44.8**	
**Experience with caring for the severely ill:**				
Ves	435	64.5	35.5	0.555
No	563	62.7	37.3	
**End-of-life care experience:**				
Yes	446	**67.6**	**32.4**	**0.020**
No	552	**60.5**	**39.5**	
**Marital status:**				
Single	219	**71.7**	**28.3**	**>0.001**
Married	529	**64.3**	**35.7**	
Extra-marital cohabitation	123	**67.5**	**32.5**	
Divorced/seperated/widowed	126	**42.9**	**57.1**	
**Persons in household:**				
Living alone	172	**53.5**	**46.5**	**>0.001**
2 persons	288	**58.7**	**41.3**	
3 or more persons	540	**69.4**	**30.6**	
**Number of children:**				
No children	708	**60.7**	**39.3**	**0.011**
1 child	137	**67.2**	**32.8**	
2 or more children	155	**72.9**	**27.1**	
**Self-rated health:**				
Very good	343	67.3	32.7	0.090
Good	445	64.0	36.0	
Moderate-very poor	205	58.0	42.0	

A strong link between the attitude towards NE and *age group* was observed: the older the interviewee, the higher the approval rates. In addition, the oldest age group (75 years and older) displayed the highest overall percentage of approval (38.6%).

There is also a strong association between the *level of education* and the attitude towards NE. Rejection rates increased with the level of education, ascending to over 80% among university graduates.

The variable *political orientation* showed higher rejection rates among politically left- (67.7%) and center-oriented (66%) interviewees than in politically right-wing oriented interviewees (55.2%). By contrast, no association between *socio-cultural ideology* and attitude towards NE was detected.

Moreover, respondents with *end-of-life care experience* were more likely to reject NE than those without. In case of *experience with the care of severely ill* and *self-rated health* no significant association was found.

An increased *number of persons* in the household, however, showed to have a significant effect on the opinion about euthanasia. The higher the number of persons in the household, the more likely was a rejection of the practice. This was also observed for the variable *number of children*, albeit to a lesser extent.

### Independent predictors of attitudes

The logistic regression model explained 13.9% of the variance based on the examined variables (Table 2).

**Table 2:** **Results of binary logistic regression analyses: rejection of neonatal euthanasia, by independent variables (n = 805)**

	Rejection of neonatal euthanasia			
	Odds ratio	95% CI		P-value
		Lower	Upper	
**Sex: male (ref = female)**	0.77	0.55	1.06	0.104
**Age group (ref = 16–25 years)**				**0.021**
25-34 years	**0.49**	**0.25**	**0.98**	**0.044**
35-44 years	0.53	0.26	1.11	0.092
45-59 years	**0.30**	**0.14**	**0.63**	**0.001**
60-74 years	**0.35**	**0.16**	**0.77**	**0.009**
75 years or older	**0.23**	**0.08**	**0.66**	**0.006**
**Level of education (ref = compulsory school)**				**0.005**
Apprentice training/intermediate vocational degree	**1.64**	**1.03**	**2.62**	**0.036**
High school diploma	**1.86**	**1.05**	**3.31**	**0.034**
University	**3.29**	**1.71**	**6.34**	**>0.001**
**End-of-life care experiences (ref = no)**	**1.75**	**1.26**	**2.41**	**0.001**
**Marital status (ref = single)**				**0.048**
Married	0.96	0.54	1.70	0.876
Extra-marital cohabitation	0.70	0.38	1.30	0.254
Divorced/separated/widowed	**0.45**	**0.23**	**0.90**	**0.024**
**Persons in household (ref = living alone)**				0.087
2 persons	1.08	0.59	1.99	0.793
3 or more persons	1.57	0.88	2.80	0.127
**Constant**	2.23			0.056
**Nagelkerkes R** ^**2**^	13.9 %			

Variables excluded by backward procedure: income, socio-cultural ideology, political orientation, experience in care of severely ill, number of children in household and self-rated health.

In regression analysis, the variable *age group* showed a significant association with the rejection of NE. A lower tendency to reject was observed with increasing age (OR = 0.23, p = 0.006, 75 years and older vs. 16–25 years old). However, no significant gender effect was shown.

Much like in univariate analysis, *educational level* here also turned out to have a great effect on respondents’ attitude towards NE. Euthanasia was more frequently rejected by people with a higher educational status (OR = 3.29, p > 0.001, university vs. compulsory school). The other socio-economic variable *family income* was, however, not associated with the rejection of NE.

Regression analysis revealed that out of the two variables regarding care experience, *end-of-life care experience* had a significant association with the attitude towards NE. People with experience in the care of terminally ill were more likely to reject NE than those without this experience (OR = 1.75, p = 0.001). By contrast, *experience with the care of severely ill* had no independent effect on the rejection of NE and was therefore excluded from the model.

The variable *marital status* only showed a significant effect when comparing single with divorced (OR = 0.45, p = 0.024), thus indicating that divorced or separated persons show lower rejection rates.

## Discussion

Overall, 63.6% of the study population rejected the idea of NE for severely impaired newborns while 36.4% opted in favor or were undecided. This percentage stands in sharp contrast with approximately 30% of competent adults who do not agree with VAE, a relationship that we found in a previous analysis of the same survey data [20]. In general, there are two main arguments supporting VAE. First of all, there is the right of patient autonomy and freedom of choice. This right suggests that an autonomous adult with decisional capacity has the right to freely decide about his/her own life or death. The second argument in support of VAE is that of beneficence and individual well-being. If a patient suffers unbearably despite optimal medical care, then one may decide that the burdens outweigh the benefits of living. In the case of NE, however, the first of the two main arguments is not applicable and the second one is controversial. Neonates obviously have no decisional capacity and are unable to express their wishes. Therefore, parents and the staff of neonatal intensive care units must rely on clinical clues and interpret an infant’s behaviour in order to assess the severity of their suffering. Moreover, even with apparent suffering is the extent of the infant’s suffering, and whether or not it is unbearable, a matter of subjective evaluation [10, 21–23].

Our data and the analysis of the same sample by Stronegger et al. [20] show different determinants for the attitudes towards VAE and NE, respectively. Therefore, it can be suggested that the respective attitude might be based on different motivations and considerations. Furthermore, in the case of VAE it was suggested that cognitive convictions – such as the ideological positioning – might be strong determinants influencing the interviewees’ opinion [20]. However, this variable did not have any effect on the interviewees’ attitude towards NE, thus hinting to a more emotional reasoning concerning end-of-life decision-making for newborns.

Our analysis confirmed a strongly positive association between a higher educational level and a higher rejection rate of NE among the Austrian population whereas several American [19, 24, 25] and European [26, 27] studies concerning VAE observed a different trend: in these studies a higher educational level was associated with a lower rejection rate. This inverse trend in Austria had already been observed in prior studies [20, 28]. We should, however, bear in mind that these studies were focusing on VAE and that it is therefore debatable whether the available data are comparable to our study. A possible approach to explain this relationship might be that persons with a higher educational level are considered to have a better health awareness and a better knowledge of prenatal care. They might, therefore, be more familiar with the early detection of high-risk pregnancies and genetic testing and thus consider NE to be highly avoidable by a more widespread use of prenatal prevention.

One of the factors that consistently correlated with the attitude towards NE was age. The only available cohort study investigating the effect of the age and birth cohort on the attitude towards VAE suggests that people mostly stick to their opinion over life [29]. Thus it can be supposed that age effects observed in cross-sectional studies concerning end-of-life attitudes are primarily birth cohort effects. Our study, however, observed a severe shift in the attitudes of the different age groups. The rejection rate among the youngest age group was twice as high as the rate observed in the oldest age group. This might be due, among other reasons, to the growing emotional value and role children play for their parents today and to the ever-decreasing number of children per family [30].

Some bioethicists have argued that parents could use the Groningen Protocol as a means to escape from the unwanted burden of caring for an impaired child [12]. Our data suggest that end-of-life care experience is positively associated with the rejection of NE. This could be an indication that both an increased willingness to give care and an open mind towards suffering would lead to an increased rejection of NE.

In recent years, the question of legalizing neonatal euthanasia along the lines indicated by the Groningen protocol has been very controversially discussed in the medical ethics literature. Approval and disapproval seem to be almost balanced when referring to the number of expressed opinions. Concerning the general public in Austria, a clear reluctance to accept legalization seems to prevail and it may increase even further. Therefore, if physicians involved in neonatal care would intend to introduce regulations such as the Groningen Protocol, strong and comprehensible arguments would be needed in a first step to gain wider public acceptance.

### Strengths and limitations

This study presents the first-ever statement, derived from a large representative sample, about the opinion on NE among the general Austrian population. However, there are some limitations:

First of all, attitude was measured using simplified self-rating questions instead of indicators that might have given more genuine information. Especially the attitude toward NE was assessed by only one single question, instead of by validated psychometrical scales that have been scarcely used in the research on end-of-life attitudes to date. Second, the inquired variables were restricted in number. Hence, other potential determinants (such as religiosity, ethnicity, ideology, or other personal characteristics) might be underrepresented in the study.

## Conclusion

The present study examined whether the idea of applying euthanasia to severely impaired infants is acceptable among a representative sample of the general Austrian population. The majority judged NE (i.e. the administration of a drug with the purpose of ending a patient’s life) as being unacceptable. Our results have shown that age (resp. birth cohort), educational level, and end-of-life care experience are strongly associated with a higher tendency to reject NE. However, given the increasing levels of rejection of NE among the younger generations and among people with a higher educational level, it cannot be precluded that the rejection rate might in future increase even further, rather than decrease.

## References

[1] van der Heide A, Onwuteaka-Philipsen BD, Rurup ML, Buiting HM, van Delden JJ, Hanssen-de Wolf JE, Janssen AG, Pasman HR, Rietjens JA, Prins CJ, Deerenberg IM, Gevers JK, van der Maas PJ, van der Wal G (2007). End-of-life practices in the Netherlands under the Euthanasia Act. N Engl J Med.

[2] Cuttini M, Casotto V, de Vonderweid U, Garel M, Kollee LA, Saracci R (2009). Neonatal end-of-life decisions and bioethical perspectives. Early Hum Dev.

[3] van der Heide A, van der Maas PJ, van der Wal G, de Graaff CL, Kester JG, Kollée LA, de Leeuw R, Holl RA (1997). Medical end-of-life decisions made for neonates and infants in the Netherlands. Lancet.

[4] Verhagen AE (2013). The Groningen Protocol for newborn euthanasia; which way did the slippery slope tilt?. J Med Ethics.

[5] Verhagen E, Sauer PJ (2005). The Groningen Protocol–euthanasia in severely ill newborns. N Engl J Med.

[6] Verhagen A, Sauer P (2005). End-of-life decisions in newborns: an approach from the Netherlands. Pediatrics.

[7] Verhagen AA, Dorscheidt JH, Engels B, Hubben JH, Sauer PJ (2009). End-of-life decisions in Dutch neonatal intensive care units. Arch Pediatr Adolesc Med.

[8] Jotkowitz A, Glick S, Gesundheit B (2008). A case against justified non-voluntary active euthanasia (the Groningen Protocol). Am J Bioeth.

[9] de Vries MC, Verhagen AA (2008). A case against something that is not the case: the Groningen Protocol and the moral principle of non-maleficence. Am J Bioeth.

[10] Chervenak FA, McCullough LB, Arabin B (2009). The Groningen Protocol: Is it necessary? Is it scientific? Is it ethical?. J Perinat Med.

[11] Appel JM (2009). Neonatal euthanasia: why require parental consent?. J Bioethical Inquiry.

[12] Lindemann H, Verkerk M (2008). The Groningen Protocol. Hastings Cent Rep.

[13] Manninen B (2006). A case for justified non-voluntary active euthanasia: exploring the ethics of the Groningen Protocol. J Med Ethics.

[14] Manninen B (2008). Revisiting justified nonvoluntary euthanasia. Am J Bioeth.

[15] Thomas FP, Beres A, Shevell MI (2006). “A Cold Wind Coming”: Heinrich Gross and Child Euthanasia in Vienna. J Child Neurol.

[16] Cuttini M, Casotto V, Kaminski M, de Beaufort I, Berbik I, Hansen G, Kollee L, Kucinskas A, Lenoir S, Levin A, Orzalesi M, Persson J, Rebagliato M, Reid M, Saracci R (2004). Should euthanasia be legal? An international survey of neonatal intensive care units staff. Arch Dis Child Fetal Neonatal Ed.

[17] Teisseyre N, Vanraet C, Sorum PC, Mullet E (2010). The acceptability among lay persons and health professionals of actively ending the lives of damaged newborns. Monash Bioeth Rev.

[18] Teisseyre N, Vanraet C, Sorum PC, Mullet E (2011). The acceptability among lay persons and health professionals of actively ending the lives of damaged newborns. Monash Bioeth Rev.

[19] Moulton BE, Hill TD, Burdette A (2006). Religion and Trends in Euthanasia Attitudes Among US Adults, 1977–2004. Sociological Forum.

[20] Stronegger WJ, Burkert NT, Grossschaedl F, Freidl W (2013). Factors associated with the rejection of active euthanasia: a survey among the general public in Austria. BMC Med Ethics.

[21] Kon AA (2007). Neonatal euthanasia is unsupportable: the Groningen protocol should be abandoned. Theor Med Bioeth.

[22] Kon AA (2008). We cannot accurately predict the extent of an infant’s future suffering: the Groningen Protocol is too dangerous to support. Am J Bioeth.

[23] Saigal S, Tyson J (2008). Measurement of Quality of Life of Survivors of Neonatal Intensive Care: Critique and Implications. Semin Perinatol.

[24] Jorgenson DE, Neubecker RC (1980). Euthanasia: a national survey of attitudes toward voluntary termination of life. OMEGA.

[25] DeCesare MA (2000). Public attitudes toward euthanasia and suicide for terminally ill persons: 1977 and 1996*. Biodemography Soc Biol.

[26] Cohen J, Marcoux I, Bilsen J, Deboosere P, van der Wal G, Deliens L (2006). European public acceptance of euthanasia: socio-demographic and cultural factors associated with the acceptance of euthanasia in 33 European countries. Soc Sci Med.

[27] Verbakel E, Jaspers E (2010). A comparative study on permissiveness toward euthanasia religiosity, slippery slope, autonomy, and death with dignity. Public Opin Q.

[28] Suarez-Almazor ME, Belzile M, Bruera E (1997). Euthanasia and physician-assisted suicide: a comparative survey of physicians, terminally ill cancer patients, and the general population. J Clin Oncol.

[29] Leinbach RM (1993). Euthanasia attitudes of older persons a cohort analysis. Res Aging.

[30] Hoffman LW (1975). The value of children to parents and the decrease in family size. Proc Am Philos Soc.

[CR31] The pre-publication history for this paper can be accessed here:http://www.biomedcentral.com/1472-6939/15/74/prepub

